# Environmental tobacco smoke exposure and diabetes in adult never-smokers

**DOI:** 10.1186/1476-069X-13-74

**Published:** 2014-09-25

**Authors:** Ikenna C Eze, Emmanuel Schaffner, Elisabeth Zemp, Arnold von Eckardstein, Alexander Turk, Robert Bettschart, Christian Schindler, Nicole Probst-Hensch

**Affiliations:** Department of Epidemiology and Public Health, Swiss Tropical and Public Health Institute, Socinstrasse 57, 4002 Basel, Switzerland; University of Basel, Basel, Switzerland; Institute of Clinical Chemistry, University Hospital, Zurich, Switzerland; Zürcher Höhenklinik Wald, Wald-Faltigberg, Faltigberg-wald, Switzerland; Lungenpraxis Hirslanden Klinik Aarau, Aarau, Switzerland

**Keywords:** Passive smoke, Type 2 diabetes, Cross-sectional study, Respiratory obstruction, Never-smokers

## Abstract

**Background:**

Active smoking has been linked to type 2 diabetes mellitus (T2DM) but only few recent studies have shown environmental tobacco smoke (ETS) to be associated with DM in never-smokers. We assessed the association between long term ETS exposure and DM, and explored effect modifications of this association in our sample.

**Methods:**

We analysed 6392 participants of the Swiss study on air pollution and lung and heart diseases in adults (SAPALDIA). We used mixed logistic regression models to assess the cross-sectional association between ETS and DM. Selected variables were tested for effect modification and several sensitivity analyses were performed, mostly treating participants’ study area as a random effect.

**Results:**

The prevalence of DM and ETS in the sample was 5.5% and 47% respectively. There were 2779 never-smokers with 4% diabetes prevalence. Exposure to ETS increased risk of DM in never-smokers by 50% [95% confidence interval (CI): 1.00, 2.26], and we observed a positive dose–response relationship between ETS exposure level and DM in never-smokers. Associations were strengthened (more than three-folds) by older age and chronic obstructive pulmonary disease, and were stronger in post-menopausal, obese, hypertriglyceridaemic and physically inactive participants. Estimates of association were robust across all sensitivity analyses (including inverse probability weighting for participation bias and fixed-effect analysis for study area). ETS had no substantial associations in current and ex-smokers in our study.

**Conclusions:**

We found a positive association between ETS exposure and DM in never smokers. Additional longitudinal studies involving biomarkers are needed to further explore underlying mechanisms and susceptibilities.

**Electronic supplementary material:**

The online version of this article (doi:10.1186/1476-069X-13-74) contains supplementary material, which is available to authorized users.

## Introduction

Diabetes contributes significantly to global disability-adjusted life years lost. Type 2 diabetes mellitus (T2DM) comprises >90% of global diabetes and its age-adjusted prevalence and incidence are steadily increasing [1].

Smoking has been established as a risk factor for incident T2DM [2]. There has been interest in the health effects of environmental tobacco smoke (ETS), which contains >4000 chemical compounds that partly overlap with compounds inhaled in active smoke [3]. ETS is still common and creates substantial health costs; for instance Switzerland spends an estimated 419 million Swiss Francs in health costs due to ETS exposure [4]. ETS is produced at different temperatures and in different forms [mainstream smoke and side smoke] [3]. Multiple chemical components of ETS occur in higher concentration in side-stream than inhaled cigarette smoke [3]. These compounds are generally inflammatory, causing blood vessel injuries and endothelial dysfunction [5, 6]. ETS also elevates plasma fibrinogen concentrations (an acute phase plasma protein and a biomarker for cardiorespiratory and metabolic diseases) to a higher level than active smoke [7]. ETS was associated with reduced insulin sensitivity [8], glucose intolerance [9] and metabolic syndrome [10]. Few studies have linked ETS to incident [11] and prevalent DM [12] in never-smokers, even in a dose–response manner [11] but no study has explored differential susceptibilities in detail. Studying never-smokers is important as the ETS effects are not intermixed with effects of previous or current active smoking. Identification of modifying factors or susceptible subgroups will help in identifying potentially causal pathways involved in the relationship between ETS and DM in never-smokers.

We therefore assessed the association between chronic ETS exposure and prevalent DM in an adult Swiss cohort, and explored possible modifying factors in this association in never-smokers.

## Methods

### Study Population

The Swiss Cohort Study on Air Pollution and Lung and Heart Diseases in Adults (SAPALDIA) included 9651 participants aged 18–65 years at baseline (1991) [13, 14]. The first follow-up examination in 2002 involved 8047 participants who underwent detailed computer- based interviews and extensive health examinations. This examination also included non-fasting blood sampling into a biobank [14]. The sample for the present study was 6392 participants aged 29–73 years, with complete data on all relevant variables, drawn from this first follow-up study. Ethical clearance for the SAPALDIA study was obtained from the Review Boards of participating Cantons.

### Definition of DM

Information on DM was not obtained at baseline therefore precluding us from studying incident diabetes. At the first follow-up health interview, participants were asked: “do you have diabetes?” and “was it diagnosed by a doctor?” Participants were also asked about their medication history. Non-fasting blood samples were analysed for random glucose level and HbA1c, in order to identify undiagnosed cases. We classified participants as having diabetes if they met one or more of the following criteria: self-reported, physician-diagnosed diabetes; history of taking anti-diabetic medication in the past month; random blood glucose (RBG) >11.1 mmol/L; and HbA1c concentration >0.065. HbA1c concentration was assessed only in participants with RBG >6.1 mmol/L.

### Assignment of individual exposures

At baseline and first follow-up health examination, participants were asked if they were active smokers, former smokers or never smokers respectively. Long-term smoking status was defined for participants based on their active smoking status at baseline and follow-up. Participants were defined as never-smokers if they reported to have never smoked both at baseline and follow-up; current smokers if they reported current smoking at follow-up (regardless of smoking status at baseline) and ex-smokers if they reported to be former smokers regardless of smoking status at baseline. ETS exposure was defined as regular exposure to ETS in the year before baseline and/or follow-up. We then created 6 smoking categories from the long-term smoking status and ETS exposure status of the participants (never-smoker/no ETS; never-smoker/ETS; ex-smoker/no ETS; ex-smoker/ETS; current smoker/no ETS; current smoker/ETS). Participants were also asked if they had been exposed to ETS at home or elsewhere (including workplace, bars, restaurants etc.); the mean number of smokers they were exposed to, and mean number of hours/day of these exposures at home, and elsewhere. These allowed us assess any differences in effect of exposure at home and outside home (considering smoking bans in some public places in Switzerland), and to compare our findings with previous studies. From these, we computed the mean hours/day of ETS exposure for each participant and categorized them into 0, >0 < 3 and ≥3 hours/day. This categorization was done because about 50% of those who reported ETS exposure reported 3 hours/day. Also this categorization has been used in previous SAPALDIA publications [15], and ensures internal validity within the SAPALDIA study. All smoking and ETS questions were derived from validated questionnaires and harmonized with the European Community Respiratory Health Survey [16].

### Potential Confounders

We selected potential confounders for this study based on literature review and plausibility. Selected confounders include sex, age, body mass index (BMI; kg/m^2^), educational level (≤9 years, 9–13 years, >13 years), neighbourhood socio-economic index (obtained from a principal component analysis involving educational level and occupation of household heads, median rent and number of persons living in a household [17]), vigorous physical activity (defined as participation in activities that makes one sweat or out of breath; <0.5, 0.5-2, >2 hours/week), consumption of citrus fruits (including juice), other fruits (including juice) and raw vegetables [including salad/juice; (<1, 1–3, >3 days/week, respectively)], consumption of alcohol (including beers, wines, liquors and spirits; never, ≤once, >once/day). We considered occupational exposure to vapours, gases, dusts or fumes (yes/no) and pack-years smoked. Since ETS contributes to particulate matter concentration [18, 19], and studies have previously shown a positive association between PM and diabetes [20, 21], we additionally considered mean ambient PM_10_ exposure (μg/m^3^) from baseline up until follow-up as a potential confounder.

Estimates of annual home outdoor levels of particulate matter <10 μm in diameter (PM_10_) were obtained for each participant and each year between baseline and first follow-up based on their address histories and inter/extrapolations of validated dispersion model estimates for the year 1990 and 2000 at all residential sites, using a spatial resolution of 200 × 200 m. Annual means (between baseline and follow-up) were inter/extrapolated using trends from monitoring stations nearest to participants’ residences. Validation and inter/extrapolation were done using data from the Swiss air pollution monitoring network [22].

As potential effect modifiers, we considered sex, age (≤50, >50 years], BMI (≤25; 25–29, ≥30 kg/m^2^), educational level, physical activity, and the following binary variables: hypertension (self-reported, or systolic blood pressure ≥ 140 mmHg and/or diastolic blood pressure ≥ 90 mmHg); chronic obstructive pulmonary disease (COPD; forced expiratory volume in 1 second, FEV_1_/forced vital capacity, FVC >0.7); high non-fasting triglyceride (>1.52 mmol/L); low HDL-cholesterol (high density lipoprotein ≤1.51 mmol/L) and high hs-CRP (high sensitivity C-reactive protein; >1.0 mmol/L). In women, menopausal status (post- vs. pre-) was also considered. All of these variables were associated with diabetes at first follow-up. Statistical Analyses.

We summarized the participants’ characteristics according to smoking categories. We calculated proportions for the categorical variables and mean/standard deviation for the continuous variables.

We fitted a mixed logistic unadjusted regression model to test interaction between smoking status and ETS exposure in their association with diabetes, to justify the classification of participants to their respective smoking categories. We fitted another model with tobacco exposure in 6 categories (generated from the combination of active smoking and ETS status) and added the potential confounders in an incremental manner. This enabled observing the effect of each of the potential confounders on the association between ETS exposure and DM for each smoking category. Our fully-adjusted model included participants’ age, sex, educational status, occupational exposure to vapour, dusts and fumes, active smoking status, smoked pack-years, alcohol consumption, consumption of raw vegetables, citrus fruits and other fruits, PM_10_ exposure and body mass index. We did not consider co-morbidities and blood markers in our fully-adjusted model (they may lie in the causal pathway of the ETS-diabetes association), but we explored their effects by adding them separately to the fully-adjusted model.

We introduced interaction terms between the smoking category - ETS in never-smokers - and pre-selected potential effect modifiers to the fully-adjusted model one by one and noted the p-values of the interaction terms. In other models, we estimated separate effects of this smoking category for the several compared groups.

We performed several sensitivity analyses. We adjusted for missing information and potential participation bias using inverse probability weighting. We excluded participants with any self-reported heart disease. We excluded participants who reported diabetes medication use before baseline assessment. We restricted diabetes definitions by only applying one of the criteria at a time and excluded cases identified by “complementary” definitions from respective analyses. We applied models ignoring study area and treating study area as a fixed effect. We created smoking categories based on only the baseline smoking and ETS data; collected 10 years prior to data obtained for diabetes classification at follow-up, and performed similar incremental adjustment for potential confounders.

To assess dose–response relationships, we limited the analysis to never-smokers and tested the association between mean hours/day (categories) of ETS exposure and prevalent diabetes by adding the potential confounding variables also in an incremental manner.

All analyses were performed with STATA data analysis software version 12 (STATA Corporation, Texas).

## Results

The prevalence of ETS in the study population was 46.8% and 34% in never-smokers. We identified 315 cases of diabetes. In never-smokers, ETS exposure was higher in females, younger participants, and participants with lower educational level, lower neighbourhood socio-economic index and lower physical activity, lower HDL, higher hs-CRP and higher diabetes rates (Table 1). Similar trends were observed for ex-smokers and current smokers. Current smokers had longer duration of exposure to ETS than never- and ex-smokers. ETS exposure in current smokers was associated with higher triglyceride levels and higher smoking pack-years (Table 1). Additional file 1: Table A1 shows the baseline characteristics of SAPALDIA cohort participants based on whether they were included in the present analysis or not. While most characteristics were significantly different, the proportion of diabetes cases was the same in both groups.

**Table 1:** **Characteristics of study population**

Characteristic (%)	Never smokers; No ETS	Never smokers; ETS	Ex-smokers; No ETS	Ex-smokers; ETS	Current Smokers; No ETS	Current Smokers; ETS
N = 6392	1834	945	1119	902	448	1144
Females	61	58	45	42	45	47
Educational level: ≤ 9 years	6	7	5	6	7	6
10-13 years	63	67	64	66	62	71
>13 years	30	26	32	28	30	24
Occupational exposure to gases/dusts/ fumes	31	47	36	55	40	54
Mean hours/day exposed to ETS at home: 0	100	55	100	55	100	34
<3	0	39	0	39	0	54
≥3	0	6	0	6	0	12
Mean hours/day exposed to ETS elsewhere: 0	100	55	100	55	100	34
<3	0	34	0	33	0	51
≥3	0	11	0	13	0	16
Vigorous physical activity: <0.5 h/wk	35	42	40	40	44	45
0.5-2 hours/week	38	32	35	30	33	30
>2 hours/week	27	26	31	30	24	25
Alcohol intake: Never	11	11	6	8	7	9
≤ once/day	84	82	86	77	83	77
> once/day	4	6	8	15	10	15
Consumption of vegetables: Never	0	1	1	0	1	1
≤3 days/week	17	19	17	18	20	23
>3 days/week	83	80	82	82	79	76
Consumption of citrus fruits: Never	7	7	9	9	10	11
≤3 days/week	57	52	57	57	59	55
>3 days/week	36	41	34	34	31	34
Consumption of other fruits: Never	1	1	1	1	2	4
≤3 days/week	28	31	29	36	41	42
>3 days/week	71	68	70	63	57	54
Menopause^a^	64	55	63	54	62	47
Diabetes cases	3	5	6	7	5	4
Hypertension cases	35	34	44	38	35	31
COPD cases	16	16	22	20	25	25
High triglyceride (>1.52 mmol/l)	45	45	54	54	51	54
High HDL-cholesterol (>1.51 mmol/l)	51	47	46	43	41	35
High C-reactive protein (>1.0 mg/l)	47	47	49	52	56	44
Mean (SD)						
Age (years)	52.6(11.9)	49.8(12.7)	56.1(9.8)	52.7(10.8)	52.9(10)	48.9(10.7)
BMI (kg/m^2^)	25.6(4.3)	26.2(4.7)	26.1(4.3)	26.7(4.4)	25.5(3.9)	25.3(4.5)
Area socio-economic index	64.6(9.7)	62.3(10.3)	65.3(9.8)	61.8(10.4)	64.7(9.9)	61.8(10.3)
Home outdoor PM_10_ (μg/m^3^)	21.9(7.3)	22.6(7.4)	21.9(7.3)	22.7(7.7)	23.6(7.5)	22.6(7.5)
Pack-years of smoking^b^	0(0)	0(0)	7.2(17.8)	13(27.5)	14.7(27.1)	20(28.4)

ETS: environmental tobacco smoke; BMI: body mass index; COPD: chronic obstructive pulmonary disease; PM_10_: particulate matter <10 μm in diameter. HDL: high density lipoproteins; HDL divided at its sample mean whereas CRP and triglycerides were divided at their sample median. ^a^N = 2958. ^b^Median (IQR).

We found higher odds of DM in ex-smokers (OR: 1.29; 95% CI: 0.87, 1.92) and current smokers (OR: 1.37; 95% CI: 0.80, 2.36) compared to never-smokers. Since we found a significant interaction between ETS exposure and smoking in their association with DM (p = 0.013), we separately estimated the effect of ETS exposure in never-smokers, ex-smokers and current smokers.

Exposure to ETS increased the odds of diabetes by 50% (95% CI: 0, 126%) in never smokers. There was a positive association in ex-smokers and no sizable association in current smokers (Table 2). Odds of diabetes generally increased by >20% in all categories after adjusting for age and sex and remained fairly stable until adjustment for BMI (Table 2). Adjusting for hypertension, triglyceridaemia, HDL and hs-CRP levels did not substantially change the effect estimates (Additional file 1: Table A2). We observed a positive dose–response relationship between level of ETS exposure and DM in never-smokers. This was substantial only for home ETS exposure. The respective odds of diabetes for ETS exposure >0 < 3 hours/day and ≥3 hours/day at home were 1.01 (95% CI: 0.54, 1.88) and 2.62 (95% CI: 1.04, 6.62) compared to no ETS exposure at home (Table 3).

**Table 2:** **Association between ETS-exposure status and diabetes mellitus according to smoking status**

	ETS (yes vs. no) in never smokers OR (95% CI)	ETS (yes vs. no) in ex-smokers OR (95% CI)	ETS (yes vs. no) in current smokers OR (95% CI)
Unadjusted	1.54 (1.05, 2.27)	1.02 (0.71, 1.45)	0.80 (0.48, 1.35)
Adjusted for age and sex	1.86 (1.26, 2.76)	1.26 (0.87, 1.82)	1.07 (0.63, 1.82)
+ socio-economic status	1.81 (1.22, 2.69)	1.21 (0.84, 1.75)	1.05 (0.62, 1.78)
+ lifestyle characteristics^a^	1.75 (1.17, 2.60)	1.19 (0.81, 1.73]	1.06 (0.62, 1.82)
+ home outdoor PM_10_	1.73 (1.16, 2.57)	1.17 (0.81, 1.71)	1.06 (0.62, 1.81)
+ body mass index	1.50 (1.00, 2.26)	1.07 (0.73, 1.57)	0.93 (0.54, 1.61)

Socio-economic status includes educational attainment and area-level socio-economic position; ^a^include alcohol consumption, smoking pack-years, work exposure to dust gas and fumes, citrus fruits, other fruits and raw vegetables, and physical activity. OR: odds ratio. OR values represent % increase in diabetes prevalence for exposure to ETS in each smoking group. CI: confidence interval. PM_10_: particulate matter <10 μm in diameter. Area was treated as a random effect in all models. + indicates additional adjustment. N = 6392 at all levels of adjustment.

**Table 3:** **Dose–response relationship between ETS exposure and DM in never-smokers**

	ETS exposure >0 < 3 hours/day at home ^a^OR (95% CI)	ETS exposure ≥3 hours/day at home ^a^OR (95% CI)	ETS exposure >0 < 3 hours/day elsewhere ^b^OR (95% CI)	ETS exposure ≥3 hours/day elsewhere ^b^OR (95% CI)
Unadjusted	1.06 (0.60, 1.85)	3.35 (1.46, 7.69)	1.47 (0.87,2.47)	0.98(0.35,2.72)
Adjusted for age and sex	1.41 (0.79, 2.51)	3.51 (1.48, 8.33)	1.77 (1.03,3.03)	1.55(0.54,4.44)
+ socio-economic status	1.33 (0.75, 2.38)	3.44 (1.45, 8.14)	1.69 (0.98,2.92)	1.47(0.51,4.22)
+ lifestyle characteristics^c^	1.26 (0.70, 2.28)	2.83 (1.18, 6.82)	1.55 (0.89,2.69)	1.40(0.48,4.09)
+ home outdoor PM_10_	1.27 (0.70, 2.30)	2.81 (1.17, 6.75)	1.55 (0.89,2.70)	1.41(0.49,4.09)
+ body mass index	1.01 (0.54, 1.88)	2.62 (1.04, 6.62)	1.25 (0.70,2.24)	1.31(0.43,4.01)

^a^estimates are compared with no ETS exposure at home. ^b^estimates are compared with no ETS exposure elsewhere. Socio-economic status includes educational attainment and area-level socio-economic position; ^c^include alcohol consumption, smoking pack-years, work exposure to dust gas and fumes, citrus fruits, other fruits and raw vegetables, and physical activity. OR: odds ratio. OR values represent % increase in diabetes prevalence for exposure to ETS compared to the reference group. CI: confidence interval. PM_10_: particulate matter <10 μm in diameter. Area was treated as a random effect in all models. + indicates additional adjustment. N = 2779.

On an ordinal scale, the risk of diabetes increased by 13% (95% CI: 0.97, 1.31) and 5% (0.96, 1.15) per hour of ETS exposure at home and elsewhere respectively.

Since we found a substantial association between ETS exposure and DM only in never-smokers, we limited the effect modification analysis to this group. The association between ETS and DM in never smokers was highly strengthened by older age and COPD (Table 4). Associations were also strengthened (albeit less strongly) by female sex, obesity, physical inactivity, hypertension, high serum triglyceride and low serum HDL, and post-menopausal status in women (Table 4).

**Table 4:** **Patterns of susceptibility to ETS in association with diabetes mellitus, in never smokers**

Variable	Categories	ETS (yes vs. no) in never-smokers.OR (95% CI)
Age	≤ 50 years	0.32 (0.07, 1.60)
	> 50 years	1.69 (1.10, 2.60)
	Interaction	0.05
Sex	Males	1.23 (0.65, 2.35)
	Females	1.71 (1.01, 2.92)
	Interaction p-value	0.443
BMI (kg/m^2^)	< 25	1.15 (0.42, 3.19)
	25-29	1.24 (0.68, 2.27)
	≥30	1.97 (1.12, 3.46)
	Interaction p-value	0.261
Hypertension	No	1.23 (0.56, 2.73)
	Yes	1.64 (1.01, 2.66)
	Interaction p-value	0.545
COPD (FEV1/FVC <0.7)	No	1.18 (0.75, 1.87)
	Yes	4.55 (1.69, 12.3)
	Interaction p-value	0.015
Educational level	≤ 9 years	1.38 (0.44, 4.31)
	>9 years	1.52 (0.98, 2.36)
	Interaction	0.254
Vigorous physical activity	<0.5 hours/week	1.88 (1.09, 3.19)
	≥0.5 hours/week	1.05 (0.62, 1.79)
	Interaction p-value	0.160
Triglyceride level (mmol/l)	≤ 1.52	0.89 (0.38, 2.10)
	> 1.52	1.85 (1.13, 3.02)
	Interaction p-value	0.149
HDL level (mmol/l)	≤ 1.51	1.59 (0.96, 2.61)
	> 1.51	1.34 (0.61, 2.94)
	Interaction p-value	0.718
C-reactive protein (mg/l)	≤1.0	1.71 (0.83, 3.56)
	>1.0	1.47 (0.89, 2.45)
	Interaction p-value	0.742
Menopause	No	0.56 (0.07, 4.33)
	Yes	2.14 (1.13, 4.05)
	Interaction p-value	0.217

OR: odds ratio, CI: confidence interval; COPD: chronic obstructive pulmonary disease, FEV_1_: forced expiratory volume in 1 second, FVC: forced vital capacity. HDL: high density lipoproteins. OR values represent % increase in prevalent diabetes for ETS exposure in never smokers in each category. Triglyceride and C-reactive protein were grouped by its median value while HDL was grouped by its mean value. Area was treated as a random effect in all models. Group-specific estimates were obtained from a single model. P-values of interaction terms (between exposure and potential effect modifiers) were obtained from a separate model.

The effect estimates remained robust and positive across most sensitivity analyses. Adjusting for participation bias, ignoring study area and treating study area as a fixed effect did not sizably change the odds of diabetes (Additional file 1: Table A3). Exclusion of participants with self-reported heart disease or using restricted definitions of diabetes based on self-reported diagnosis or medication intake substantially increased the odds of diabetes (Additional file 1: Table A3). Exclusion of subjects who reportedly used anti-diabetic medication at the baseline assessment and defining diabetes only using the blood markers marginally reduced the odds of diabetes (Additional file 1: Table A3). Defining exposure categories using only the baseline smoking and ETS status did not sizably change the results (Additional file 1: Table A4).

## Discussion

We found a positive association between ETS exposure and diabetes among never smokers in the SAPALDIA cohort. This association was strengthened by age and respiratory obstruction.

We also found a positive dose–response relationship which was substantial for ETS exposure at home. We observed some threshold effect for ETS exposure outside the home. This points towards a non-linear association but on further exploration, we found the likelihood ratio test comparing the model with categorical hours of ETS exposure with the model with ordinal hours of ETS exposure to be insignificant (P-value > 0.2) for both exposure settings. Thus we may not reject the hypothesis of a linear dose–response relationship, particularly as these dose–response relationships were observed using only three categorical data points.

Major characteristics and findings of previous studies (which reported mixed findings) on this association in never-smokers are summarized in Additional file 1: Table A5. While most reported a positive association between ETS exposure and DM risk [9, 12, 23–27], others did not [10], despite controlling for major DM risk factors. A recent meta-analysis of six studies reported pooled risk of diabetes to be 1.21 (95% CI: 1.07, 1.38) in never-smokers. Few studies explored effect modification by age [23], sex [9, 23, 25], race [9], BMI/obesity [25, 26], pre-diabetes status [24] and living with a regular/occasional smoker [26]. No study explored modification by physical activity, social status, menopause and co-morbidities. Unlike our study, Ko et al. did not find any effect modification by age [23]. Our finding of a stronger effect in women agrees with other studies [9, 23]. This might be because women get more intense ETS exposure than men (they comprise >60% of never-smokers in our study) and/or hormonal differences impacting on the metabolism [28, 29]. We found no substantial effect modification by BMI/obesity. Unlike Lajous et al. [26], our results suggest effects to be stronger among the obese. Stronger effects were reported for white race [9], pre-diabetic subjects [24], and people living with a regular smoker [26].

We found a positive dose–response relationship only for home ETS exposure which appeared to be stronger in men. This may indicate that exposures at home tend to be more intense and sustained than exposures elsewhere, which would be consistent with the general decline in smoking in public places in Switzerland over the past years [30]. Lajous et al. reported a contrary finding where they demonstrated a positive association for exposures outside the home, which might be because they could not assign home exposures to a large proportion of their participants [26]. Ko et al. [23] demonstrated a positive dose–response relationship between ETS exposure and incident T2DM both at work and home in their population. The differences between ETS at home and ETS elsewhere should be interpreted with care as we observed similar dose–response trends in exhaled carbon monoxide (CO; a marker of recent exposure to tobacco smoke) levels in both settings (Additional file 1: Table A6).

None of these studies explored the effect of ETS in the three smoking groups (never-, ex- and current smokers). Some studies either combined never- and ex-smokers [10, 12, 25] or studied ETS exposure only in never smokers [9]. One study explored effect of ETS exposure in never and ex-smokers, but not in current smokers [24]. This makes comparison of the various results difficult.

As suggested by Iso et al. [7], ETS elevates plasma fibrinogen levels. Our finding of effect modification by age and COPD are consistent with a fibrinogen-related pathway, since plasma fibrinogen increases with age [31] and is a biomarker associated with COPD risk [32].

ETS has been shown to cause adipose tissue inflammation and alter lipid profiles [33]. Holay et al. demonstrated higher levels of serum lipids among active and passive smokers [5], and Xie et al. showed an association between ETS and metabolic syndrome, including its individual lipid components [10]. We found stronger associations with ETS among people with high triglyceride, low HDL-cholesterol level and obesity, which also points to a pathway involving lipids. Particulate matter concentration is influenced by ETS and has also been shown to cause adipose tissue inflammation, endothelial dysfunction and impaired insulin signalling in animal models [34].

Hormonal changes in menopause may enhance visceral adiposity, high levels of adipokines and tumour necrosis factor-alpha [35, 36], which are risk factors for insulin resistance. Our results showed a >2-fold risk of DM for ETS exposure in post-menopausal compared to pre-menopausal women. Henkin et al. demonstrated reduced insulin sensitivity on ETS exposure both in never- and ex-smokers, which was more pronounced in females [8].

This analysis draws from the large database of the SAPALDIA study with detailed socio-demographic, lifestyle and health characteristics of participants. The ETS exposures were assigned based on detailed questions regarding the smoking habits of participants and the people around them. We observed little or no inconsistencies in self-reported smoking status at baseline and follow-up, for instance, none of the never-smokers at follow-up reported smoking at baseline. Our adjustment for socio-economic (SE) differences did not only include individual educational attainment, but also SE status at neighbourhood-level. Moreover, we controlled for potential confounding effects from exposure to ambient air pollution, using home outdoor levels of PM_10_, and from exposure at work, by using an indicator variable for occupational exposure to dusts, gases and fumes. Our outcome definition was based on an algorithm attempting to minimise misclassification. We assessed effect modification by selected variables to help understand potential pathways involved in the observed associations.

We used a cross-sectional design and all estimates of ETS exposure were self-reported which poses a risk for bias. Ideally, cotinine concentrations are measured to objectively assess ETS exposure. Nevertheless we expect some reliability in the self-reported ETS exposure as they have been shown to moderately correlate with serum cotinine measures [37, 38]. Also, we observed a positive gradient in mean expired CO levels stratified by categories of self-reported hours of ETS exposure (Additional file 1: Table A6). We did not have any information on family history of diabetes and were unable to differentiate adult type 1 diabetes (T1DM) from T2DM in our population. We expect a small bias due to T1DM since most of the global diabetes burden and adult diabetes diagnosis are due to T2DM [39]. We did not have information on time of diagnosis for many DM cases but about 90% of those on DM medication reported intake after baseline examination. We explored this in a sensitivity analysis by excluding the few cases that reported diabetes medication intake before baseline examination. Our results might have been affected by participation bias since only 67% of the original cohort participants could be included in the analyses. Therefore, we conducted a sensitivity analysis using inverse probability weighting. The fact that these results were very similar to the original ones does not rule out participation bias completely but reduces concerns to some extent.

Estimates of association remained robust across most sensitivity analyses apart from exclusion of reported diabetes prior to baseline. This could mean that excluded cases (reportedly diagnosed before baseline) may have had a longer exposure duration compared to the other cases. Also, they are older and we have shown association to be stronger in older people. We cannot entirely exclude under-diagnosis or under- or over-reporting of diabetes in the absence of a clinical diabetes assessment in all participants. Unless this misclassification is related to ETS, we would expect it to bias associations towards null.

Our findings in ex-smokers and current smokers are not surprising, especially for current smokers because of their constant exposure to their own active smoke. It would be necessary to further explore this in ex-smokers, taking into account the time between smoking cessation and ETS exposure assessment. A hypothesis is that effects of ETS increase with longer duration between smoking cessation and ETS exposure assessment.

## Conclusion

We found a positive association between ETS exposure and DM in never smokers, which was significantly stronger in older subjects and in subjects with COPD. High quality longitudinal studies including various biomarkers are needed to explore these effect modifications in more detail.

## Electronic supplementary material

Additional file 1: **Title and description of data.** **Table A1.** Characteristics of included and zexcluded participants. **Table A2.** Additional adjustment for hypertension and blood markers. **Table A3.** Sensitivity analyses of ETS-DM association in never smokers. **Table A4.** Sensitivity analyses with baseline variables **Table A5.** Previous studies ETS and risk of DM in never-smokers. **Table A6.** Mean expired carbon monoxide levels by categories of self-reported ETS exposure in never-smokers. (DOCX 37 KB)

## Abbreviations

BMI: Body mass index
CO: Carbon monoxide
COPD: Chronic obstructive pulmonary disease
DM: Diabetes mellitus
ETS: Environmental tobacco smoke
FEV1: Forced expiratory volume in one second
FVC: Forced vital capacity
HDL: High density lipoprotein
hs-CRP: High sensitivity C-reactive protein
PM10: Particulate matter with diameter less than 10 micrograms
RBG: Random blood glucose
SAPALDIA: Swiss cohort study on air pollution and lung and heart diseases in adults
SE: Socio-economic
T1DM: Type 1 diabetes mellitus
T2DM: Type 2 diabetes mellitus.
