# Supplementary material for: Environmental tobacco smoke exposure and diabetes in adult never-smokers
Source: Environ Health. 2014 Sep 25;13:74. doi: 10.1186/1476-069X-13-74 (PMC4192739; doi:10.1186/1476-069X-13-74)
Supplement: Supplementary file 1 — Additional file 1: Title and description of data. Table A1. Characteristics of included and zexcluded participants. Table A2. Additional adjustment for hypertension and blood markers. Table A3. Sensitivity analyses of ETS-DM association in never smokers. Table A4. Sensitivity analyses with baseline variables Table A5. Previous studies ETS and risk of DM in never-smokers. Table A6. Mean expired carbon monoxide levels by categories of self-reported ETS exposure in never-smokers. (DOCX 37 KB) [file 12940_2014_783_MOESM1_ESM.docx]

**ADDITIONAL FILE**

**Environmental Tobacco Smoke Exposure and Diabetes in Adult Never-Smokers.**

Ikenna C. Eze^1,2^, Emmanuel Schaffner^1,2^, Elisabeth Zemp^1,2^, Arnold von Eckardstein^3^ , Alexander Turk^4^, Robert Bettschart^5^, Christian Schindler ^1,2^ and Nicole Probst-Hensch ^1,2^.

^1^Swiss Tropical and Public Health Institute, Basel, Switzerland

^2^University of Basel, Switzerland

^3^Institute of Clinical Chemistry, University Hospital, Zurich, Switzerland

^4^Zürcher Höhenklinik Wald, Wald-Faltigberg, Switzerland

^5^Lungenpraxis Hirslanden Klinik Aarau, Switzerland.

Table of Contents

Table A1. Characteristics of included and excluded participants…………………………...2

Table A2 Additional adjustment for hypertension and blood markers......................................4

Table A3 Sensitivity analyses of ETS-DM association in never-smokers …………………..5

Table A4 Sensitivity analyses with baseline variables………………………………………..6

Table A5 Previous studies ETS and risk of DM in never-smokers…………………………..7

Table A6 Mean expired carbon monoxide levels by categories of self-reported ETS exposure in never-smokers.……………………………………………………………………………..11

Table A1: Characteristics of included and excluded participants

BMI: body mass index; PM_10_: particulate matter <10µm in diameter. HDL: high density lipoproteins; HDL divided along its sample mean whereas CRP and Triglycerides were divided along their sample median. ^a^N=2958; ^b^Median; ^c^Median test.

| Characteristic [%] | Number of exclusions/ inclusions | Excluded participants | Included participants | P-value [chi-square] |
| --- | --- | --- | --- | --- |
| Females | 704/3278 | 54.6 | 51.3 | 0.027 |
| Educational level: ≤ 9 years | 1277/6392 | 18.5 | 0.6 | <0.001 |
| 10-13 years | 1277/6392 | 64.2 | 65.6 | 0.342 |
| >13 years | 1277/6392 | 17.3 | 28.2 | <0.001 |
| Occupational exposure to gases/dusts/ fumes | 3259/6392 | 1.4 | 27.5 | <0.001 |
| Smoking status: Never | 615/2779 | 37.2 | 43.5 | <0.001 |
| Former | 516/2021 | 31.2 | 31.6 | 0.732 |
| Current | 524/1592 | 31.7 | 24.9 | <0.001 |
| ETS exposure | 3259/6392 | 48.9 | 46.8 | 0.040 |
| Mean hours/day exposed to ETS at home: 0 | 264/4802 | 86.8 | 75.1 | <0.001 |
| <3 | 35/1338 | 11.5 | 20.9 | <0.001 |
| ≥3 | 5/252 | 1.6 | 3.9 | 0.042 |
| Mean hours/day exposed to ETS elsewhere: 0 | 263/4800 | 86.5 | 75.1 | <0.001 |
| <3 | 30/1197 | 9.9 | 18.7 | <0.001 |
| ≥3 | 11/395 | 3.6 | 6.2 | 0.068 |
| Vigorous physical activity: <0.5 h/week | 64/2475 | 45.4 | 38.7 | 0.108 |
| 0.5-2 hours/week | 44/2154 | 31.2 | 33.7 | 0.536 |
| >2 hours/week | 33/1736 | 23.4 | 27.6 | 0.272 |
| Alcohol intake: Never | 18/585 | 10.6 | 9.2 | 0.522 |
| ≤ once/day | 129/5229 | 75.9 | 81.8 | 0.049 |
| > once/day | 23/578 | 13.5 | 9.0 | 0.045 |
| Consumption of vegetables: Never | 0/40 | 0 | 0.6 | 0.298 |
| ≤3 days/week | 29/1203 | 16.9 | 18.8 | 0.516 |
| >3 days/week | 143/5149 | 83.1 | 80.6 | 0.397 |
| Consumption of citrus fruits: Never | 12/536 | 7.0 | 8.4 | 0.537 |
| ≤3 days/week | 90/3588 | 53.0 | 56.1 | 0.408 |
| >3 days/week | 68/2268 | 40.0 | 35.5 | 0.320 |
| Consumption of other fruits: Never | 2/115 | 1.2 | 1.8 | 0.556 |
| ≤3 days/week | 53/2120 | 31.5 | 33.2 | 0.660 |
| >3 days/week | 113/4157 | 67.3 | 65.0 | 0.550 |
| Menopause | 122/2958 | 53.3 | 58.5 | 0.253 |
| Diabetes cases | 1036/6392 | 4.9 | 4.9 | 0.994 |
| Hypertension cases | 1035/6392 | 13.3 | 6.8 | <0.001 |
| COPD cases | 184/6392 | 23.4 | 21.7 | 0.596 |
| High triglyceride level (>1.52 mmol/l) | 205/6111 | 49.8 | 49.8 | 0.984 |
| High HDL level (>1.51 mmol/l) | 204/6098 | 40.7 | 44.9 | 0.230 |
| High C-reactive protein (>1.0 mg/l) | 205/6111 | 54.1 | 49.9 | 0.233 |
| Mean (SD) |  |  |  | T-test |
| Age (years) | 1288/6392 | 51.5(12.1) | 52.2(11.4) | 0.038 |
| BMI (kg/m^2^) | 206/6392 | 26.6(5.4) | 25.9(4.4) | 0.024 |
| Neighbourhood socio-economic index | 1598/6392 | 62.7(10.7) | 63.5(10.1) | 0.005 |
| Home outdoor PM_10_ (µg/m^3^) | 1184/6392 | 22.7(6.9) | 22.3(7.4) | 0.143 |
| Pack-years of smoking^b^ | 1104/6392 | 0.7(20) | 0(16) | 0.411^c^ |

Table A2: Additional adjustment for hypertension and blood markers

^a^adjusted for age, sex, body mass index, educational level, area socio-economic index, occupational exposure to gases, dusts and fumes, pack-years of smoking, dietary habits, physical activity and home outdoor PM_10_. Hypertension defined as self-reported, physician-diagnosed and/or anti-hypertensive medication use and/or blood pressure >140/90 mmHg. High triglyceride defined as triglyceride >sample median (1.52 mmol/l). HDL: high-density lipoproteins. High HDL defined as HDL > sample mean (1.51mmol/l). hs-CRP: high-sensitivity C-reactive protein. High hs-CRP defined as hs-CRP > sample median (1.0 mmol/l). Area was treated as a random effect in all models. OR: odds ratio. OR values represent % increase in diabetes prevalence for exposure to ETS in each smoking group. CI: confidence interval. + indicates additional adjustment. N= 6111.

|  | ETS (yes vs. no) in never smokers  OR [95% CI] | ETS (yes vs. no) in ex-smokers  OR[95% CI] | ETS (yes vs. no) in current smokers  OR [95% CI] |
| --- | --- | --- | --- |
| Fully adjusted model ^a^ | 1.54 (1.01, 2.35) | 1.10 (0.74, 1.63) | 0.95 (0.54, 1.66) |
| + adjusted for hypertension | 1.57 (1.03, 2.39) | 1.14 (0.76, 1.70) | 0.93 (0.53, 1.63) |
| + adjusted for high triglyceride | 1.56 (1.02, 2.37) | 1.14 (0.77, 1.71) | 0.91 (0.52, 1.60) |
| + adjusted for high HDL | 1.52 (0.99, 2.33) | 1.17 (0.78, 1.76) | 0.91 (0.52, 1.62) |
| + adjusted for high hs-CRP | 1.53 (1.00, 2.34) | 1.17 (0.78, 1.75) | 0.92 (0.52, 1.63) |

Table A3: Sensitivity analyses of ETS-DM association in never-smokers

All models were adjusted for age, sex, body mass index, educational level, area socio-economic index, occupational exposure to gases, dusts and fumes, dietary habits, physical activity and home outdoor PM_10_. OR: odds ratio. OR: values represent % increase in diabetes prevalence for exposure to ETS in each smoking group. CI: confidence interval.

|  | N (Cases) | ETS (yes vs. no) in never smokers  OR (95% CI) |
| --- | --- | --- |
| IPW adjustment for participation bias | 6392 (315) | 1.52 (1.01, 2.29) |
| Exclusion of subjects with self-reported heart disease | 5951 (259) | 1.67 (1.07, 2.60) |
| Exclusion of subjects who reported diabetes medication use before baseline assessment | 6373 (296) | 1.47 (0.97, 2.25) |
| Diabetes defined as self-reported, physician diagnosis | 6306 (229) | 1.79 (1.12, 2.86) |
| Diabetes defined as self-reported diabetes medication | 6224 (147) | 2.75 (1.55, 4.81) |
| Diabetes defined as non-fasting serum glucose≥11.1 mmol/l or HbA1c≥0.065 | 6298 (221) | 1.40 (0.85, 2.29) |
| Fully adjusted fixed effect model | 6392 (315) | 1.48 (0.98, 2.23) |
| Fully adjusted model ignoring study area | 6392 (315) | 1.50 (1.00, 2.25) |

Table A4: Sensitivity analyses with baseline variables

Socio-economic status includes educational attainment and area-level socio-economic index; ^a^ include smoking pack-years, work exposure to dust gas and fumes. OR: odds ratio. OR values represent % increase in diabetes prevalence for exposure to ETS in each smoking group. CI: confidence interval. PM10: particulate matter <10µm in diameter. Area was treated as a random effect in all models. + indicates additional adjustment.

|  | N | ETS (yes vs. no) in never-smokers  OR (95% CI) | ETS (yes vs. no) in ex-smokers  OR (95% CI) | ETS (yes vs. no) in current smokers  OR (95% CI) |
| --- | --- | --- | --- | --- |
| Unadjusted | 6392 | 1.55 (1.06, 2.25) | 1.13 (0.72, 2.85) | 0.65 (0.43, 0.97) |
| Adjusted for age and sex | 6392 | 1.83 (1.24, 2.69) | 1.23 (0.78, 1.95) | 0.86 (0.56, 1.30) |
| + socio-economic status | 6392 | 1.79 (1.21, 2.63) | 1.17 (0.74, 1.86) | 0.84 (0.56, 1.28) |
| + lifestyle characteristics^a^ | 6278 | 1.77 (1.20, 2.60) | 1.24 (0.78, 1.99) | 0.89 (0.57, 1.39) |
| + home outdoor PM_10_ | 6278 | 1.77 (1.21, 2.61) | 1.25 (0.78, 2.00) | 0.90 (0.58, 1.40) |
| + body mass index | 6251 | 1.49 (0.99, 2.25) | 1.11 (0.68, 1.82) | 0.91 (0.57, 1.45) |

Table A5: Previous studies ETS and risk of DM in never-smokers

OR: odds ratio. HR: hazards ratio. CI: confidence interval. T2DM: type 2 diabetes mellitus. OGTT: oral glucose tolerance test. ETS: environmental tobacco smoke. BMI: body mass index.

| Source | Population (N), Age group, Follow-up duration | Assessment of ETS exposure | Outcome definition | Confounder adjustment | Fully-adjusted effect estimate (95% CI) | Effect modification | Limitations |
| --- | --- | --- | --- | --- | --- | --- | --- |
| Ko et al. 2011, Ansung/Ansan, Korea | 10,038 participants of the Korean Genome and Epidemiology Study aged 40-69 years.  Follow-up: 6 years | Self-reported exposure to ETS defined as having situations of direct inhalation of smoke from other smokers in the home or at workplace at baseline. | Incident T2DM defined as fasting serum glucose ≥126mg/dl or 2-hour oral glucose tolerance test (OGTT) ≥200mg/dl or self-reported diabetes treatment during follow-up. | Baseline age, sex, residential area, education level, alcohol consumption, waist circumference, regular exercise, hypertension, total cholesterol, homeostatic model assessment of insulin resistance [HOMA-IR] and glucose tolerance status. | HR: 1.41 (1.16, 1.70) | Risk of T2DM not different by sex and age (data not reported) | Probable false reporting of medication intake and/or smoking status. |
| Kowall et al. 2010, Augsburg, Germany | 885 participants of the KORA S4/F4 study aged 55-74 years.  Follow-up: 7 years | Self-reported exposure to ETS in the household or at workplace at baseline. | Incident T2DM defined as validated self-reported physician-diagnosed diabetes or anti-diabetic medication intake or diagnosis by OGTT during follow-up. | Baseline age, sex, parental diabetes, socio-economic status, alcohol intake, physical activity, intake of meat and sausage, intake of salad and vegetables, intake of whole grain bread and coffee consumption. | OR: 2.5 (1.1, 5.6) | Risk of T2DM is stronger among pre-diabetic subjects (OR: 4.4; 1.5, 13.4) than normoglycemic subjects (OR: 1.3; 0.3, 6.8) | Exposure status was self-reported. |
| Vardavas et al. 2010, Cyprus and Greek Islands | 1190 participants of the MEDIS study aged 65-100 years. | Self-reported exposure to ETS defined as daily ETS exposure [≥30 minutes] and number of years of exposure | Prevalent T2DM defined as fasting plasma glucose >6.1 mmol/L or self-reported current use of glucose-lowering medication | Age, sex, smoking status, hypertension, hypercholesterolemia, family history of diabetes, physical activity, BMI, educational level, Mediterranean diet score. | OR: 1.60 (1.05; 2.51) | None assessed | Exposure to ETS was self-reported |
| Zhang et al. 2011, USA | 100526 women in the Nurses’ Health Study aged 41-54 years.  Follow-up: 24 years | Self-reported exposure to ETS defined as current regular exposure to cigarette smoke from other people at baseline. | Incident T2DM defined as self-reported physician-diagnosed diabetes in the two years preceding questionnaire administration during follow-up. | Age, race, BMI, physical activity, husband’s education, family history of diabetes, total energy intake, intake of alcohol, magnesium, calcium, vitamin D, total trans-fat, fibre from cereal, caffeine, total fat and saturated fat. | RR: 1.16 (1.00, 1.35) | None assessed | Study limited to women, who were mostly white.  Exposure to ETS was self-reported. |
| Hayashino et al. 2008, Japan | 6498 participants of the High Risk and Population Strategy for occupational Health Promotion Study aged 19-69 years  Follow-up: 4 years | Self-reported exposure to ETS in the workplace defined as current exposure to ETS without active smoking at baseline, regardless of past smoking status | Incident T2DM defined as fasting blood glucose level ≥7.0mmol/ L or random plasma glucose ≥11.1 mmol/L or treatment with hypoglycaemic medication or self-reported history of diabetes during follow-up. | Baseline age, sex, BMI, physical activity, alcohol, family history of diabetes, hypertension, health promotion intervention, frequency of sweetened beverage intake, frequency of vegetable intake, do not care about eating too much fat at all. | HR: 1.81 (1.06, 3.19) | Interaction with sex: P=0.74  Interaction with obesity: P=0.77  Interaction with health promotion intervention: P=0.087 | Exposure to ETS was self-reported. |
| Houston et al. 2006, USA | 4657 participants of the Coronary Artery Risk Development in Young Adults (CARDIA) study aged 18-30 years  Follow-up: 15 years | Self-reported exposure to passive smoke and serum cotinine level of 1-15ng/ml at baseline | Incident glucose intolerance defined as fasting serum glucose≥100 mg/dl or self-report of anti-diabetic medication intake at any point during follow-up | Age, sex, race, years of education, income, systolic blood pressure, triglycerides, alcohol consumption and smoking pack-years. | HR: 1.35 (1.06, 1.71) | Stronger effects in white men (HR: 1.66; 1.11, 2.49) and white women (HR: 1.89; 0.98, 3.64) than African-American men (HR: 1.26; 0.80, 1.97) and African-American women (HR: 0.97; 0.60, 1.56).  Interaction with race: P<0.001. | Participants made of entirely urban population. |
| Xie et al. 2009, China | 396 students and 389 parents aged 30-54 years, from 304 households | Self-reported exposure to ETS defined as being in a room or vehicle with someone who was smoking in the past seven days. | Glucose intolerance defined as fasting plasma glucose ≥100mg/dl or previously diagnosed T2DM | Age, sex, education, income, alcohol consumption and active smoking status | OR: 1.1 (0.3, 4.1) | Not assessed | ETS exposure was self-reported. |
| Lajous et al. 2013, France | 37,343 female non-smokers of the E3N cohort.  Follow-up: 15 years | Self-reported exposure to ETS during childhood and adulthood. | T2DM defined as self-reported T2DM or use of diabetic medications or hospitalization for diabetes | Baseline age, BMI, education, parental history of diabetes, body silhouette at age 8, menopausal status, physical activity, diet, treated hypertension and hypercholesterolemia. | HR for childhood exposure: 1.15 (0.99, 1.33).  HR for adulthood exposure:1.16 (1.00, 1.34) | Stronger effects for outside home exposure≥4h/day (HR: 1.45; 1.01, 2.07); living with a regular smoker (HR: 1.15; 0.95, 1.39) than an occasional smoker (HR: 1.10; 0.84, 1.44); those with BMI<25 kg/m^2^ (HR: 1.82; 1.20, 2.76) than those with BMI≥25 kg/m^2^ (HR: 1.36; 0.99, 1.89) | Self-reported ETS exposure measured only at baseline. Lack of data on parental social status. |

Table A6: Mean expired carbon monoxide levels by categories of self-reported ETS exposure in never-smokers.

Carbon monoxide (CO) values are Mean (SD); ppm: parts per million.

|  | ETS exposure at home (hours/day) | | | ETS exposure elsewhere (hours/day) | | |
| --- | --- | --- | --- | --- | --- | --- |
|  | 0 | >0<3 | ≥3 | 0 | >0<3 | ≥3 |
| Expired CO (ppm) | 2.0 (1.7) | 2.2 (1.7) | 2.4 (1.8) | 1.9 (1.7) | 2.1 (1.6) | 2.5 (1.9) |
